# Urinary IgM excretion: a reliable marker for adverse pregnancy outcomes in women with chronic kidney disease

**DOI:** 10.1007/s40620-018-0536-9

**Published:** 2018-09-11

**Authors:** Alfredo Leaños-Miranda, Inova Campos-Galicia, Karla Leticia Ramírez-Valenzuela, María Guadalupe Berumen-Lechuga, Irma Isordia-Salas, Carlos José Molina-Pérez

**Affiliations:** 1 Medical Research Unit in Reproductive Medicine, UMAE-Hospital de Ginecología y Obstetricia “Luis Castelazo Ayala”, Instituto Mexicano del Seguro Social (IMSS), Don Luis # 111, Col. Nativitas, 03500 Mexico City, DF Mexico; 2 Division of Maternal-Fetal Medicine, UMAE-Hospital de Ginecología y Obstetricia “Luis Castelazo Ayala”, Instituto Mexicano del Seguro Social (IMSS), Mexico City, Mexico; 3 Department of Obstetrics and Gynecology, Hospital General Regional No. 251 Metepec, IMSS, Metepec, Estado de México Mexico; 4 Medical Research Unit in Thrombosis, Hemostasia and Atherogenesis, H.G.R. No. 1 “Dr. Carlos Mac Gregor”, IMSS, Mexico City, Mexico

**Keywords:** Pregnancy, Chronic kidney disease, Biomarkers, Urinary IgM, Adverse outcomes

## Abstract

**Objective:**

Chronic kidney disease (CKD) pregnancies are at high risk of developing adverse outcomes. In non-pregnant subjects with CKD, higher urinary IgM levels are associated with poor renal survival and higher rates of cardiovascular deaths. In this study, we assessed whether urinary IgM levels are associated with an increased risk of adverse pregnancy outcomes (APO) in CKD pregnancies.

**Methods:**

We performed a nested case–control study within a cohort of CKD patients with singleton pregnancies attended at a tertiary care hospital. The study included 90 CKD patients who eventually developed one or more APO and 77 CKD patients who did not. Urinary IgM excretion was determined from the 24-h urine samples at enrollment by an ultrasensitive enzyme immunoassay.

**Results:**

The risk for combined APO and for preeclampsia (PE) was higher among women with urinary IgM and proteinuria levels values in the highest quartile or with CKD stages 4–5 (odds ratios, OR ≥ 2.9), compared with the lowest quartile or with CKD stage 1. Urinary IgM levels were more closely associated with the risk of either combined or specific APO (PE, preterm birth, and for having a small-for-gestational-age infant; OR ≥ 5.9) than either the degree of total proteinuria or CKD stages. Among patients with CKD stage 1, the risk of combined APO, PE, and preterm birth was higher in women with urinary IgM levels values in the highest quartile (OR ≥ 4.8), compared with the three lower quartiles, independently of proteinuria.

**Conclusion:**

In CKD pregnancies, at the time of initial evaluation, proteinuria and CKD stage are associated with increased risk of combined APO. However, urinary IgM concentrations appear to be better predictors of an adverse outcome and may be useful for risk stratification in CKD pregnancies.

## Introduction

Chronic kidney disease (CKD) is increasing worldwide, and its prevalence is estimated to be 8–16% in the general population [1]. Although CKD is associated with reduced fertility, pregnancy can occur in women with any stage of CKD [2]. Pregnancy with CKD is considered to be high risk and this disease affects 3.3% of pregnancies [3]. It is well recognized that CKD is generally associated with poor fetal and maternal outcomes. Although advances in medical and obstetric management in women with CKD has improved and the rate of adverse pregnancy outcomes (APO) has decreased in the last decades, still they have an increased frequency of APO [4–8]. Women even in the early stages of CKD have high frequency of APO compared to healthy controls [4–6], and these APO may occur in up 70% of pregnancies of women with more advanced CKD [6, 7]. Therefore, the overall risks of APO increase incrementally with the severity of CKD, and proteinuria and hypertension are modulators [6–8]. Interestingly, it has been demonstrated that the risk for APO is not merely linked to kidney function reduction, since patients with stage 1 CKD and controls differ significantly even after controlling the effects of other classic risk factors, such as proteinuria, hypertension, and systemic diseases, suggesting the presence of a baseline risk linked to CKD per se, which leads to a persistent renal damage [9]. This highlights the urgent need for identification of biomarkers related to the persistent renal damage that may aid in the risk stratification for APO in pregnant women with CKD.

In non-pregnant subjects, several factors may influence the clinical outcome of CKD such as type of kidney disease, race, gender, age, and hypertension. Moreover, the degree and type of proteinuria are a good indicator of the extent of kidney damage and are involved in the pathophysiology of disease [10]. Urinary excretion of proteins of high molecular weight, such as immunoglobulin (Ig)M has been used in differentiating glomerular diseases [11]. High urinary IgM (uIgM) excretion rather than the level of albuminuria is associated with poor renal survival in several glomerular diseases [12–15], as well as with lower survival, especially due to an increase in cardiovascular deaths [12, 16]. Furthermore, increased uIgM excretion is associated with coronary artery disease and cardiovascular events [16–18].

Nevertheless, there is no available information on the uIgM levels in pregnant women with CKD. The goal of the present study was to investigate whether differences in uIgM levels in patients with CKD may identify those patients at high risk to develop APO.

## Patients and methods

The protocol study was approved by our institute’s review board. Written informed consent was obtained from all participants. Between March 2011 and December 2017, all women with singleton pregnancies diagnosed with CKD before o during pregnancy were recruited from the Department of Maternal-Fetal Medicine of a tertiary care level hospital. Patients with systemic lupus erythematosus were excluded from the study because lupus nephritis per se has been associated with worse pregnancy outcomes for multiple reasons [19].

CKD stage was defined according to Kidney Disease: Improving Global Outcomes (KDIGO) guidelines [20]. Given the lack of consensus regarding the best marker for renal function during pregnancy, glomerular filtration rate (GFR) was calculated using the Chronic Kidney Disease Epidemiology Collaboration (CKD-EPI) formula [21] based on preconception data or serum creatinine measured at first control in pregnancy. This formula was chosen because of its wide use in several previous studies on CKD and pregnancy.

Hypertension was defined as systolic or diastolic blood pressure ≥ 140 mmHg or ≥ 90 mmHg, or antihypertensive therapy prior to 20 weeks’ gestational age. Preeclampsia (PE) was defined according to The American College of Obstetricians and Gynecology criteria [22]. PE without severe features was defined as hypertension and significant proteinuria (≥ 300 mg protein in a 24-h urine specimen or a protein to creatinine ratio ≥ 0.30 mg/mg in a random urine sample). PE with severe features was considered when either hemolysis, elevated liver enzymes, low platelet count (HELLP) syndrome, eclampsia, or PE with severe hypertension (systolic or diastolic blood pressure ≥ 160 mmHg or ≥ 110 mmHg, respectively) was present. Other parameters included, even in absence of significant proteinuria, were new-onset cerebral or visual disturbances, abnormal liver enzymes levels (to twice normal concentration), thrombocytopenia (< 100,000/µl), or pulmonary edema. Superimposed PE (i.e. with preexisting hypertension and/or proteinuria) was considered when HELLP syndrome, eclampsia, or other severe features were present; in their absence, a sudden increase in blood pressure and/or sudden increase in proteinuria accompanied by an angiogenic imbalance, as assessed by the maternal serum soluble fms-like tyrosine kinase-1/placental growth factor (sFlt-1/PlGF) ratio ≥ 85, measured by electrochemiluminescence [23, 24], was considered as superimposed PE.

Other APO included any of the following: preterm delivery (< 34 weeks of gestational age), the neonatal death (stillbirths [defined as death of a fetus] and neonatal death [defined as death of a newborn until hospital discharge]) and small-for-gestational age (SGA) infant, defined as an infant whose birth weight was below the 10th percentile.

A 24-h timed urine collection was obtained at enrollment to estimate GFR using standard creatinine clearance corrected for the body surface area and to measure total protein and IgM excretion. Samples were centrifuged, and the resulting sediment-free urine specimens were aliquoted and stored at − 80 °C until assayed. All samples were collected before clinical suspicion or diagnosis confirmation of any studied APO. Clinical and delivery outcomes were recorded to classify the patients with APO.

### Urine analysis

IgM concentrations in urine were measured by an ultrasensitive enzyme immunoassay for human IgM developed in our laboratory to measure small amounts of IgM. Polystyrene 96-well plates (Nunc, Roskilde, Denmark) were coated with 100 µl/well of polyclonal antibodies specific for human IgM (goat anti-h IgM, American Qualex, San Clemente, CA, USA) at a concentration of 1 µg/ml in 0.1 M carbonate–bicarbonate buffer, pH 9.0. Plates were incubated for 2 h at 37 °C and stored at 4 °C until used. Optimum blocking conditions for nonspecific binding were achieved using 300 µl/well of 5% skimmed milk in phosphate-buffered saline (PBS), pH 7.4, containing Tween-20 at 0.05% (PBST), for 2 h at 37 °C. After washing five times with PBST, the samples were added in duplicate at different dilutions to ascertain parallelism with the standard curve (Bethyl Laboratories, Inc., Montgomery, TX, USA). The plates were left for 1 h at 37 °C, then washed five times with PBST, and subsequently incubated with monoclonal antibodies specific for human IgM (American Qualex, San Clemente, CA, USA) at a concentration of 1 µg/ml. Following 1 h of incubation at 37 °C, the plates were washed five times with PBST and subsequently incubated with a peroxidase-conjugated goat anti-mouse serum (Dako, Carpinteria, CA, USA), at a 1:2000 dilution for 1 h at 37 °C. Plates then were washed five times with PBST before the reactions were revealed with o-phenylene diamine (Sigma, St. Louis, MO, USA). Optical density was read at 492 nm using an ELISA reader (Emax, Molecular Device). The minimal detectable quantity was 3.0 ng/ml, and the intra- and interassay coefficients of variation were 3.5 and 5.6%, respectively. Urinary protein and creatinine were measured as previously described [25].

### Statistical analysis

Differences between continuous variables were determined by the unpaired Student t test (or the Mann–Whitney U test for non-normally distributed variables). Differences between categorical variables were determined by the Chi square test with Yates continuity correction or the Fisher exact test for small samples (or the Mantel–Haenszel χ^2^ test with linear tendency for variables with > 2 categories). Association between uIgM and total protein levels, and CKD stages, and the subsequent risk of APO, was calculated. Test results were grouped into quartiles (urinary total protein and IgM excretion) and kidney disease was grouped via CKD stage; due to the clinical severity and sample size, the groups were collapsed into four groups, CKD stage 1, 2, 3 and CKD stage 4–5; and the odds ratios (ORs) and 95% confidence intervals (CIs) were calculated to assess the association between quartiles or CKD stage and the risk of APO. The lowest quartile was used as reference category for both proteinuria and IgM-uria, and for CKD stage, group 1 (CKD stage 1) was the reference group. Logistic-regression analysis was used to adjust the ORs for proteinuria, IgM-uria, and CKD stage because these variables were significantly different among the groups studied by univariate analysis, as well as for chronic hypertension considering that this variable may distinctly affect the adverse outcomes. In addition, the ORs for preterm delivery were also adjusted for PE, and for neonatal death were adjusted for gestational age at delivery. A two-tailed p < 0.05 was considered statistically significant.

### General description of the population

Of the 171 CKD patients enrolled, 4 (2.3%) were excluded for the following reasons: two patients for congenital defects, one patient for molar pregnancy, and one patient for loss to follow-up at week 30. Therefore, a total of 167 CKD pregnancies were available for final analysis. Each patient was included only once in the study. The mean maternal age was 28.9 ± 6.2 years with a median gestational age at enrollment of 21 weeks (IQR 15–25). The majority of patients were multigravida (57.9%). In 130 of 167 cases (77.8%), the diagnosis of CKD was made during pregnancy. Glomerulonephritis was the main cause of CKD (69.4%), followed by diabetes (20.4%) and tubulointerstitial (10.2%). Chronic hypertension was present in 50.9% of patients. Of the 167 patients, 59 (35.3%) had CKD stage 1, 48 (28.7%) CKD stage 2, 38 (22.8%) CKD stage 3, 15 (9.0%) CKD stage 4, and 7 (4.2%) CKD stage 5. Hemodialysis was required in four patients with CKD stage 5.

Ninety patients (53.1%) had ≥ 1 APO, including PE 66 (39.5%), SGA infant 61 (36.5%), preterm birth 53 (31.7%), and neonatal deaths 21 (12.6%). Among the 53 patients who delivered at < 34 weeks of gestation, 47 of deliveries were indicated for PE, two for fetal distress, two for preterm premature rupture of membranes, and two for spontaneous preterm labor.

### uIgM levels, proteinuria, and CKD stages are associated with the occurrence of APO

Tables 1 and 2 show the comparison between patients who developed combined APO or PE and those who did not. A significant trend towards higher frequency for combined APO was observed across CKD stages: CKD stage 1, 19/59 (32.2%); CKD stage 2, 25/48 (52.1%); CKD stage 3, 25/38 (65.8%); and CKD stages 4–5, 21/22 (95.5%) (p < 0.001). A similar trend was observed for PE: CKD stage 1, 14/59 (23.7%); CKD stage 2, 19/48 (31.3%); CKD stage 3, 17/38 (44.7%); and CKD stages 4–5, 16/22 (72.7%) (p < 0.001). Tubulointerstitial nephropathy patients had a lower frequency of combined APO and PE compared to patients with glomerular disease or diabetic nephropathy.

**Table 1:** Clinical, laboratory, and demographic characteristics of pregnant women with CKD according to the occurrence of combined adverse pregnancy outcomes

Variable	Combined adverse pregnancy outcomes		p
	No (n = 77)	Yes (n = 90)	
Maternal age, years, mean ± SD	29.0 ± 6.2	28.9 ± 6.2	0.92
Body mass index, mean ± SD	27.1 ± 5.1	26.9 ± 5.6	0.90
Primigravida, n (%)	30 (39.0)	41 (45.6)	0.48
Gravidity, median (IQR)	2 (1–2)	2 (1–2)	0.54
Miscarriage, median (IQR)	0 (0–1)	0 (0–0)	0.42
Prior preeclampsia, n (%)	6 (7.8)	13 (14.4)	0.27
Smoking, n (%)	7 (9.1)	6 (6.7)	0.77
Gestational age at sampling, week, median (IQR)	21 (14.4–25)	21 (15–25)	0.94
Chronic hypertension, n (%)	35 (45.5)	50 (55.6)	0.25
Diabetes mellitus, n (%)	11 (14.3)	23 (25.6)	0.11
Diabetes mellitus type 1, n (%)	3 (3.9)	6 (6.7)	0.66
Diabetes mellitus type 2, n (%)	8 (10.4)	17 (18.9)	0.19
Hypothyroidism, n (%)	8 (10.4)	6 (6.7)	0.56
CKD stage (KDIGO)			
1	40 (51.9)	19 (21.1)	
2	23 (29.9)	25 (27.8)	< 0.001
3	13 (16.9)	25 (27.8)	
4–5	1 (1.3)	21 (23.3)	
Type of renal disease			
Glomerulonephritis, n (%)	51 (66.2)	65 (72.2)	0.50
Diabetic, n (%)	11 (14.3)	23 (25.6)	0.11
Tubulointerstitial, n (%)	15 (19.5)	2 (2.2)	< 0.001
Creatinine clearance rate, ml/min/1.73 m^2^, mean ± SD	104 ± 49	65 ± 45	< 0.001
Proteinuria, mg/24 h, median (IQR)	763 (392–2087)	2694 (1180–4633)	< 0.001
Serum creatinine, mg/dl, mean ± SD	0.85 ± 0.43	1.59 ± 1.17	< 0.001
Urinary IgM, µg/24 h, median (IQR)	3.98 (0.0–17.26)	81.00 (29.62–173.44)	< 0.001
Gestational age at delivery, median (IQR)	38 (37–38)	33 (30–35)	< 0.001
Infant’s birth weight, g, mean ± SD	2816 ± 326	1478 ± 678	< 0.001
Cesarean delivery, n (%)	65 (84.4)	83 (92.2)	0.14
Adverse pregnancy outcomes			
Combined adverse pregnancy outcomes	0	90 (100)	
Delivery at < 34 weeks, n (%)	0	53 (58.9)	< 0.001
Preeclampsia, n (%)	0	66 (73.3)	< 0.001
Small for gestational age infant, n (%)	0	61 (67.8)	< 0.001
Neonatal deaths, n (%)	0	21 (23.3)	< 0.001

*CKD* chronic kidney disease, *KDIGO* Kidney Disease Improving Global Outcomes, *SD* standard deviation, *IQR* interquartile range

**Table 2:** Clinical, laboratory, and demographic characteristics of pregnant women with CKD according to the occurrence of preeclampsia

Variable	Preeclampsia		p
	No (n = 101)	Yes (n = 66)	
Maternal age, years, mean ± SD	29.5 ± 6.4	28.0 ± 5.8	0.13
Body mass index, media ± DE	27.0 ± 4.9	27.0 ± 6.2	0.97
Primigravida, n (%)	38 (37.6)	33 (50.0)	0.16
Gravity, median (IQR)	2 (1–2)	2 (1–2)	0.25
Miscarriage, median (IQR)	0 (0–0)	0 (0–0)	0.59
Prior preeclampsia, n (%)	9 (8.9)	10 (15.2)	0.32
Smoking, n (%)	7 (9.1)	6 (6.7)	0.83
Gestational age at sampling, week, median (IQR)	20 (14.0–24.6)	22 (17.0–26.0)	0.14
Chronic hypertension, n (%)	51 (50.5)	34 (51.5)	0.98
Diabetes mellitus, n (%)	19 (18.8)	15 (22.7)	0.68
Diabetes mellitus type 1, n (%)	6 (5.9)	3 (4.5)	0.92
Diabetes mellitus type 2, n (%)	13 (12.9)	12 (18.2)	0.47
Hypothyroidism, n (%)	10 (9.9)	4 (6.1)	0.56
CKD stage (KDIGO)			
1	45 (44.6)	14 (21.2)	
2	29 (28.7)	19 (28.8)	< 0.001
3	21 (20.8)	17 (25.8)	
4–5	6 (5.9)	16 (24.2)	
Type of renal disease			
Glomerulonephritis, n (%)	66 (65.4)	50 (75.8)	0.21
Diabetic, n (%)	19 (18.8)	15 (22.7)	0.68
Tubulointerstitial, n (%)	16 (15.8)	1 (1.5)	0.01
Creatinine clearance rate, ml/min/1.73 m^2^, mean ± SD	95 ± 50	65 ± 46	< 0.001
Proteinuria, mg/24 h, median (IQR)	1048 (455–2479)	2744 (1180–4923)	< 0.001
Serum creatinine, mg/dl, mean ± SD	1.03 ± 0.78	1.59 ± 1.14	< 0.001
Urinary IgM, µg/24 h, median (IQR)	8.70 (0.0–38.12)	80.59 (24.95–173.44)	< 0.001
Gestational age at delivery, median (IQR)	37.1 (35–38)	33 (30–35)	< 0.001
Infant’s birth weight, g, mean ± SD	2470 ± 771	1522 ± 656	< 0.001
Delivery at < 34 weeks, n (%)	15 (14.9)	39 (59.1)	< 0.001
Cesarean delivery, n (%)	86 (85.1)	62 (93.9)	0.13
Small for gestational age infant, n (%)	14 (13.9)	47 (71.2)	< 0.001
Neonatal deaths, n (%)	8 (7.9)	13 (19.7)	0.04

For other abbreviations, see Table 1

*SD* standard deviation, *IQR* interquartile range

Compared to patients without combined APO or PE, patients with these outcomes had higher serum creatinine levels, lower baseline creatinine clearance, as well as a higher baseline excretion of both total protein and IgM (p < 0.001). Patients who developed combined APO or PE had lower gestational age at delivery, delivered infants with lower birth weights, and had a greater proportion of preterm birth, SGA infants, and neonatal deaths than those patients without these APO (p ≤ 0.04). Although there was an overall increase in the frequency of cesarean section (88.6%), there was no significant difference between patients who developed combined APO or PE and patients who did not develop these outcomes (p ≥ 0.13).

### uIgM levels, proteinuria, and CKD stages and the risk of APO

Logistic regression analyses were used to determine the association between quartiles of 24-h urinary excretion of proteins and IgM, and CKD stages and risk of combined and specific APO (Table 3). The risk of combined APO was progressively higher as uIgM level quartiles increased. Similarly, for women in the third and fourth quartiles of uIgM the ORs for PE, preterm birth, or SGA infant progressively increased, suggesting that the relationship between values of this biomarker and risk of these outcomes has a linear dose–response pattern. Women in the third and fourth quartiles for total proteinuria only exhibited higher risk for a combined APO and PE, with the ORs remaining similar across quartiles (ORs 5.7 and 5.2; and 3.3 and 2.9, respectively). For CKD stages, there was an increased risk of combined APO in patients with stage 2 and 3 (ORs 3.6 and 3.4), whereas this risk was markedly higher in those patients with stage 4–5 (OR 41.0). Women with stage CKD 4–5 only exhibited higher risk for PE and SGA infant (ORs 4.5 and 4.4, respectively). With regard to the risk of neonatal deaths, there were no significant differences according to quartiles of urinary excretion of IgM and proteins, or CKD stages.

**Table 3:** Risk of adverse pregnancy outcomes according to interquartile range of urinary IgM and proteinuria levels and CKD stages in patients with CKD

Variable		Quartile 1	Quartile 2	Quartile 3	Quartile 4
Urinary IgM, µg/24 h	Range	≤ 3.26	3.27–21.35	21.36–93.72	> 93.72
Proteinuria, mg/24 h	Range	≤ 595	596–1536	1537–3690	> 3690
CKD stage	KDIGO	1	2	3	4–5

Adverse pregnancy outcome	Variable	Quartile 1 KDIGO 1 n/N OR (95% CI) Single variable **Multivariable**	Quartile 2 KDIGO 2 n/N OR (95% CI) Single variable **Multivariable**	Quartile 3 KDIGO 3 n/N OR (95% CI) Single variable **Multivariable**	Quartile 4 KDIGO 4–5 n/N OR (CI 95%) Single variable **Multivariable**
Combined	Urinary IgM	6/42	13/42	31/42	40/41
		Reference	2.7 (0.9–8.0) **4.1** (**1.1**–**15.7**)^a^	16.9 (5.6–51.0) **21.2** (**5.4**–**84.1**)^a^	240.0 (27.6–2090) **192.2** (**18.4**–**2007**)^a^
	Proteinuria	9/42	20/42	29/42	32/41
		Reference	3.3 (1.3–8.7) **1.7** (**0.4**–**6.7**)^a^	8.2 (3.1–21.9) **5.7** (**1.4**–**23.5**)^a^	13.0 (4.6–37.0) **5.5** (**1.3**–**23.5**)^a^
	KDIGO	19/59	25/48	25/38	21/22
		Reference	2.3 (1.0–5.0) **3.5** (**1.1**–**10.9**)^a^	4.0 (1.7–9.6) **3.4** (**1.1**–**12.9**)^a^	44.2 (5.5–354) **38.1** (**3.6**–**399.4**)^a^
Preeclampsia	Urinary IgM	5/40	11/42	20/42	30/42
		Reference	2.4 (0.8–7.7) **2.0** (**0.6**–**6.4**)^a^	6.4 (2.1–19.4) **4.6** (**1.5**–**14.3**)^a^	17.5 (5.5–55.4) **11.0** (**3.2**–**38.1**)^a^
	Proteinuria	7/42	13/42	22/42	24/41
		Reference	2.2 (0.8–6.4) **1.3** (**0.4**–**4.3**)^a^	5.5 (2.0–15.1) **3.4** (**1.1**–**10.7**)^a^	7.1 (2.5–19.6) **2.9** (**1.0**–**9.3**)^a^
	KDIGO	14/59	19/48	17/38	16/22
		Reference	2.1 (0.9–4.8) **2.3** (**0.6**–**6.0**)^a^	2.6 (1.1–6.3) **1.6** (**0.6**–**4.6**)^a^	8.6 (2.8–26.1) **4.5** (**1.3**–**15.7**)^a^
Preterm birth < 34 weeks	Urinary IgM	4/42	7/42	21/42	22/41
		Reference	1.9 (0.5–7.1) **1.9** (**0.5**–**7.1**)^b^	9.5 (2.9–31.4) **7.3** (**2.1**–**25.4**)^b^	11.0 (3.3–36.5) **5.9** (**1.6**–**21.7**)^b^
	Proteinuria	6/42	14/42	13/42	21/41
		Reference	3.0 (1.1–8.0) **1.7** (**0.5**–**5.9**)^b^	2.7 (0.9–8.0) **1.4** (**0.4**–**4.8**)^b^	6.3 (2.2–18.2) **2.8** (**0.8**–**9.4**)^b^
	KDIGO	12/59	15/48	15/38	12/22
		Reference	1.8 (0.7–4.3) **1.7** (**0.6**–**4.6**)^b^	2.6 (1.0–6.3) **2.0** (**0.7**–**5.7**)^b^	4.7 (1.6–13.5) **3.1** (**0.9**–**10.6**)^b^
Small of gestational age infant	Urinary IgM	2/42	8/41	24/40	27/40
		Reference	2.3 (0.6–8.3) **2.7** (**0.7**–**10.5**)^a^	14.3 (4.3–47.7) **14.1** (**3.8**–**52.3**)^a^	19.7 (5.8–67.1) **15.4** (**3.9**–**60.9**)^a^
	Proteinuria	4/42	13/40	23/42	21/39
		Reference	2.9 (0.9–8.6) **1.6** (**0.4**–**6.0**)^a^	7.3 (2.5–20.9) **4.6** (**1.3**–**16.4**)^a^	7.0 (2.4–20.4) **2.4** (**0.7**–**8.7**)
	KDIGO	12/58	19/48	16/37	14/20
		Reference	2.1 (0.9–4.7) **2.4** (**0.9**–**6.8**)^a^	2.4 (0.9–5.8) **1.5** (**0.5**–**4.6**)^a^	7.3 (2.4–22.7) **4.4** (**1.2**–**16.3**)^a^
Neonatal death	Urinary IgM	1/42	3/42	10/42	7/41
		Reference	3.2 (0.3–32.4) **1.3** (**0.01**–**2633**)^c^	13.1 (1.6–107.9) **2.7** (**0.01**–**1166**)^c^	8.6 (1.0–73.8) **7.9** (**0.01**–**5812**)^c^
	Proteinuria	2/42	4/42	6/42	9/41
		Reference	2.1 (0.4–12.2) **0.3** (**0.01**–**66.1**)^c^	3.3 (0.6–17.6) **0.6** (**0.01**–**78.9**)^c^	5.6 (1.1–27.9) **0.1** (**0.01**–**20.7**)^c^
	KDIGO	3/59	4/48	5/38	9/22
		Reference	1.7 (0.4–8.0) **10.8** (**0.3**–**348**)^c^	2.8 (0.6–12.6) **4.7** (**0.06**–**399**)^c^	12.9 (3.1–54.5) **2.9** (**0.1**–**82.8**)^c^

*CI* confidence interval, *OR* odds ratio

^a^Odds ratios were adjusted for proteinuria, urinary IgM, CKD stages, and chronic hypertension

^b^The odds ratios for preterm birth < 34 weeks were also adjusted for preeclampsia

^c^The odds ratios for neonatal deaths were also adjusted for gestational age at delivery

### uIgM excretion and the risk of APO among patients with CKD stage 1

To determine the effect of eventual differences in uIgM excretion on the association with the development of APO, a further analysis was carried out, limited to stage 1 CKD patients. For this analysis, patients were divided into two groups according to quartiles based on the distribution of uIgM levels among all stage 1 CKD patients. The first group included 30 patients whose values for uIgM levels fell within the three lowest quartiles, and the second group comprised the 29 remaining patients in the highest quartile (> 8.95 µg/24 h). The demographic, clinical, and obstetric characteristics of the patients are shown in Table 4. As expected, serum creatinine levels and baseline creatinine clearance were similar in both groups. Proteinuria was significantly higher among patients in the higher quartile for uIgM levels in comparison to the lower three quartiles. Patients in the higher quartile for uIgM levels had lower gestational age at delivery, delivered infants with lower birth weights, and had a greater proportion of preterm birth, APO, and PE than patients in the three lower quartiles (p ≤ 0.015). Although the frequency of cesarean section, SGA infant and neonatal deaths was higher in patients in the higher quartile than in patients in the three lower quartiles, the differences were not statistically significant (p ≥ 0.051).

**Table 4:** Clinical, laboratory, and demographic characteristics of pregnant women with CKD stage 1 according to urinary IgM excretion

Variable	Quartiles of urinary IgM		p
	Q1–3 0–8.95 µg/24 h (n = 30)	Q4 > 8.95 µg/24 h (n = 29)	
Maternal age, years, mean ± SD	30.5 ± 5.9	30.0 ± 6.3	0.74
Body mass index, mean ± SD	28.8 ± 6.5	30.5 ± 4.9	0.42
Primigravida, n (%)	8 (57.1)	7 (43.8)	0.82
Gravity, median (IQR)	2 (1–3)	2 (2–2)	0.49
Miscarriages, median (IQR)	0 (0–1)	0 (0–0)	0.32
Prior preeclampsia, n (%)	1 (3.3)	3 (10.3)	0.35
Smoking, n (%)	6 (20.0)	1 (3.4)	0.10
Gestational age at sampling, median (IQR)	20.5 (13.0–24.0)	21.0 (16.0–24.0)	0.77
Chronic hypertension, n (%)	14 (46.7)	14 (48.3)	0.89
Diabetes mellitus, n (%)	7 (23.3)	5 (17.2)	0.80
Diabetes mellitus type 1, n (%)	2 (6.7)	0 (0.0)	0.49
Diabetes mellitus type 2, n (%)	5 (16.7)	5 (17.2)	1.00
Hypothyroidism, n (%)	4 (13.3)	2 (6.9)	0.67
Type of renal disease			
Glomerulonephritis	20 (66.7)	24 (82.8)	0.23
Diabetic	7 (23.3)	5 (17.2)	0.75
Tubulointerstitial	3 (10.0)	0 (0.0)	0.24
Creatinine clearance rate, ml/min/1.73 m^2^, mean ± SD	143.6 ± 37.9	135.6 ± 31.3	0.38
Proteinuria, mg/24 h, median (IQR)	612.4 (385.1–1280.5)	2479.0 (1316.7–4922.8)	< 0.001
Serum creatinine, mg/dl, mean ± SD	0.61 ± 0.18	0.64 ± 0.17	0.60
Gestational age at delivery, median (IQR)	38 (37–38)	35 (33–38)	0.003
	37.2 ± 1.9	33.9 ± 5.5	0.002
Infant’s birth weight, g, mean ± SD	2779 ± 444	2081 ± 888	< 0.001
Delivery at < 34 weeks, n (%)	1 (3.3)	11 (37.9)	0.001
Cesarean delivery, n (%)	27 (90.0)	27 (93.1)	1.00
Combined adverse outcomes, n (%)	3 (10.0)	16 (55.2)	< 0.001
Preeclampsia, n (%)	3 (10.0)	11 (37.9)	0.015
Neonatal deaths, n (%)	0 (0.0)	3 (10.3)	0.11
Small for gestational age infant, n (%)	0 (0.0)	4 (13.8)	0.051

Adverse pregnancy outcome		OR (95% CI)
Combined adverse pregnancy outcome^a^	Reference	11.5 (2.6–51.0)
Preeclampsia^a^	Reference	4.8 (1.1–20.6)
Preterm birth < 34 weeks^a^	Reference	24.4 (2.6–229.9)

*SD* standard deviation, *IQR* interquartile range, *CI* confidence interval, *OR* odds ratio

^a^Odds ratios were adjusted for proteinuria and chronic hypertension

Additionally, ORs for quartiles of uIgM levels were calculated. A further adjustment for proteinuria levels was made because this variable differed significantly between groups at univariate analysis. The outcomes of SGA infant and neonatal deaths were not analyzed because of their very low frequency in both groups. Table 4 shows that the ORs were significantly high for combined APO, PE, and preterm birth (OR ≥ 4.8) among patients in the higher quartile of the CKD 1 stage patients distribution.

## Discussion

To the best of our knowledge, the present report is the first to have prospectively examined the association between basal uIgM excretion and the risk to subsequently develop APO in CKD pregnancies. In the present nested case–control study, involving a large number of pregnant women with CKD with a wide spectrum of renal disease severity and using detailed diagnostic criteria to define APO, we found that uIgM levels were significantly higher in CKD patients who eventually developed either combined APO or PE. Furthermore, in CKD patients whose uIgM levels fall within the higher quartiles, the risk of combined APO and the various individual APO, including PE, preterm birth, and SGA infant, was progressively higher as uIgM levels quartiles increased, even after taking into account the degree of proteinuria, CKD stages, and PE. Moreover, we also demonstrated that patients with CKD stage 1 whose values for uIgM excretion fall within the highest quartile have an increased risk for combined APO, as well as for PE and preterm birth compared to CKD stage 1 patients in the three lower quartiles, regardless of the degree of proteinuria, suggesting that changes in uIgM concentration effectively reflect the extent and intensity of damage to the glomerulus and, probably as well, to the systemic vascular endothelium. Further, we identified that uIgM levels are superior to the degree of proteinuria and CKD stages in predicting the risk of combined APO, as well as of other specific APOs studied.

It has been suggested that very large proteins, like IgM, with a molecular radius of 120 Å, and red blood cells are able to pass the glomerular capillary wall (GCW) through the “large pores” or “shunt pathways”. The intact GCW exhibits a very small number of these defects, and a repairing apparatus normally seals these shunts [26, 27]. Varying degrees of ultrastructural defects in glomerular basement membranes (between 15 and 200 nm in diameter) by transmission electron microscopy have been found in glomerulonephritis patients, but not in normal renal tissue [28]. Therefore, the concentration of uIgM depends on the amount of highly unselective pathways or shunts in the GCW [29], and thus reflects the severity of glomerular damage [11, 12, 14, 26]. In addition, patients with a low proteinuria selectivity index or with high uIgM excretion, exhibit a significantly high degree of fibrosis and global glomerulosclerosis. In contrast, the degree of albuminuria does not correlate to these histopathological renal biopsy findings [11, 14–16]. Likewise, it has been demonstrated that the IgM selectivity index is correlated to the development of tubulointerstitial fibrosis, suggesting that high molecular weight proteins are more damaging to the tubulus than albumin per se [14]. Taken together, these data suggest that uIgM excretion may indeed contribute to the pathogenesis of glomerular diseases and its progression, rather than representing merely a biomarker of the presence of shunt pathways in the GCW. In this vein, several studies have shown that increased uIgM excretion is a useful biomarker to characterize the glomerular diseases, and is a predictive factor for both poor renal survival and cardiovascular mortality independently of micro- or macroalbuminuria [11, 14–16]. Furthermore, it has been demonstrated that, in patients with chest pain, an increased uIgM excretion is associated with coronary artery disease and long-term cardiovascular complications, independently of the degree of albuminuria, kidney function, diabetes, and hypertension [17, 18]. These latter observations suggest that uIgM excretion reflects not only localized renal damage but also the presence of more generalized vascular endothelial dysfunction (due to atherosclerosis). In fact, it has been suggested that an increase in renal vascular resistance, due atherosclerosis, may induce ischemia and more severe structural changes in the glomeruli, including shunt pathways [16]. APO (such as PE and intrauterine growth restriction or SGA) are closely associated to generalized endothelial dysfunction due to placental malperfusion. The preexisting atherosclerotic vascular disease may also induce placental ischemia or even aggravate already existing placental dysfunction as assessed by Doppler velocimetry of the uterine artery, which in turn causes or accelerates the occurrence of these particular APO. This may explain why uIgM excretion at the time of the initial evaluation was associated with the risk of developing PE and SGA infants in our cohort of CKD pregnancies.

Previous studies have demonstrated that patients with stage 1 CKD are at higher risk than healthy women for developing APO independently of hypertension, degree of proteinuria or type of renal disease [8, 9, 30, 31], suggesting the presence of a baseline risk linked to CKD per se. In line with this idea, we found that uIgM excretion is prevalent even in patients with stage 1 CKD. Furthermore, in this group of patients, higher levels of uIgM excretion were associated with the risk of combined APO, PE, and preterm birth, independently of the degree of proteinuria and hypertension, thereby reflecting the presence of a severe and persistent renal damage. To our knowledge, the present report is the first to show that uIgM level may be a relevant biomarker of the presence of this baseline risk linked to CKD in pregnant patients and to indicate the potential for its use in clinical practice. Lastly, it has been proposed that urine IgM excretion is a marker of acute ongoing damage, while serum creatinine is more reflective of the cumulative glomerular damage [12].

Consistent with the notion that CKD pregnancies are at high risk for developing APO, and that these complications increase incrementally with the severity of renal function impairment [6, 9], we confirmed and extended a stepwise increase in the frequency of combined APO, as well as PE from stage 1 to stages 4–5 CKD. In addition, the frequencies of combined APO were similar to those found in a prior study [9]. In accordance with previous studies, we found a high frequency of PE in our cohort (39.5%), but similar to the frequency in previous studies ranging from 38.9 to 55% [32, 33]. Previous studies have reported a frequency of preterm birth ranging from 17.5 to 43% and of SGA infants ranging from 35 to 64% [9, 32, 33]. In the present study, we also found similar frequencies (32.3 and 36.5%, respectively).

The main risk factors that are associated with APO in patients with CKD include the following: renal function, degree of proteinuria, hypertension, and systemic diseases [6, 8, 9, 30–34]. In this regard, logistic regression analysis revealed that CKD stages and degree of proteinuria showed only an increased risk of combined APO and PE. However, more importantly, we found that uIgM levels markedly improved the sensitivity to predict either combined or specific APO (PE, preterm birth, and SGA infant) risk when compared with the CKD stages or degree of proteinuria. Although on univariate analysis, we found that degree of proteinuria, uIgM levels, and CKD stages were associated with the risk of neonatal deaths, when adjusting for gestational age at delivery on logistic regression analysis, there were no significant differences, suggesting that the degree of prematurity is the major determinant of neonatal mortality.

Particularly noteworthy is the fact that, in our study, a total 130 (77.8%) patients were diagnosed to have CKD during pregnancy. This contrasts with data reported in other studies, which found frequencies ranging from 17.5 to 27.5% [5, 33]. This discrepancy may stem from underdiagnosis because of the variable and subtle clinical manifestations, at least in the early phases of CKD. Thus, pregnancy provides an opportunity for early diagnosis of CKD. This highlights the need to increase awareness and provision of preconception health services in our country, as well as the importance of postpartum follow-up in all women with significant proteinuria during pregnancy, in order to confirm or rule out CKD.

The strengths of our study include a well-defined, large population of women with CKD (using criteria similar to those used previously in assessing the stage of renal disease in pregnancy) who had received the same standardized medical protocol, and the fact that all samples were collected before clinical suspicion or diagnostic confirmation of APO, which evidently avoided a selection bias. Further, we believe that the incorporation of sFlt-1/PlGF ratio in the definition of PE allowed us better to identify this disease in our patients [24]. To rule out the effects of potential confounders, the estimated ORs were adjusted for established risk factors. On the other hand, we are aware of the presence of certain limitations, including relatively late referral and a diagnosis of CKD mainly during pregnancy, precluding the analysis of other relevant outcomes, such as miscarriages; however, the frequency of combined and specific APO is in line with previous studies [9, 32, 33]. Unfortunately, we did not collect data on utero-placental flows before the development of PE, which may be considered as a limitation of this study. Lastly, we cannot generalize our findings to women with other types of renal disease, such as systemic lupus erythematosus or with other autoimmune disorders.

Finally, uncovering the mechanism of altered uIgM excretion in CKD may also provide insights into novel therapeutic options. Further prospective longitudinal studies are still needed to assess the relationship between uIgM excretion and surrogate markers of endothelial dysfunction and atherosclerosis in pregnant women with CKD.

In conclusion, our results demonstrate that uIgM levels are associated with the risk of combined and specific APO in patients with CKD. Furthermore, uIgM levels appear to be better biomarkers than the classic risk factors such as CKD stage, proteinuria, or hypertension, and may be useful for risk stratification of APO even in the early stages of CKD. Lastly, the determination of uIgM concentration is an easy assay to perform and is cheap (the cost approximately is 40 USD per 96-well plate).

## References

[1] Jha V, Garcia-Garcia G, Iseki K, Li Z (2013). Chronic kidney disease: global dimension and perspectives. Lancet.

[2] Wiles KS, Nelson-Piercy C, Bramham K (2018). Reproductive health and pregnancy in women with chronic kidney disease. Nat Rev Nephrol.

[3] Munkhaugen J, Lydersen S, Romundstad PR, Widerøe TE, Vikse BE, Hallan S (2009). Kidney function and future risk for adverse pregnancy outcomes: a population-based study from HUNT II, Norway. Nephrol Dial Transplant.

[4] Katz AI, Davison JM, Hayslett JP, Singson E, Lindheimer MD (1980). Pregnancy in women with kidney disease. Kidney Int.

[5] Abe S (1991). An overview of pregnancy in women with underlying renal disease. Am J Kidney Dis.

[6] Piccoli GB, Attini R, Vassario E (2010). Pregnancy and chronic kidney disease: a challenge in all CKD stages. Clin J Am Soc Nephrol.

[7] Williams D, Davison J (2008). Chronic kidney disease in pregnancy. BMJ.

[8] Piccoli GB, Attini R, Cabiddu G (2017). Maternal-foetal outcomes in pregnant women with glomerulonephritides. Are all glomerulonephritides alike in pregnancy?. J Autoimmun.

[9] Piccoli GB, Cabiddu G, Attini R (2015). Risk of adverse pregnancy outcomes in women with CKD. J Am Soc Nephrol.

[10] Remuzzi G, Bertani T (1998). Pathophysiology of progressive nephropathies. N Eng J Med.

[11] Tencer J, Torffvit O, Thysell H, Rippe B, Grubb A (1998). Proteinuria selectivity index based upon alpha 2-macroglobulin or IgM is superior to the IgG based index in differentiating glomerular diseases. Tech Note Kidney Int.

[12] Bakoush O, Segelmark M, Torffvit O, Ohlsson S, Tencer J (2006). Urine IgM excretion predicts outcome in ANCA-associated renal vasculitis. Nephrol Dial Transplant.

[13] Bakoush O, Tencer J, Tapia J, Rippe B, Torffvit O (2002). Higher urinary IgM excretion in type 2 diabetic nephropathy compared to type 1 diabetic nephropathy. Kidney Int.

[14] Bakoush O, Torffvit O, Rippe B, Tencer J (2001). High proteinuria selectivity index based upon IgM is a strong predictor of poor renal survival in glomerular diseases. Nephrol Dial Transplant.

[15] Tofik R, Torffvit O, Rippe B, Bakoush O (2012). Urine IgM-excretion as a prognostic marker for progression of type 2 diabetic nephropathy. Diabetes Res Clin Pract.

[16] Tofik R, Torffvit O, Rippe B, Bakoush O (2009). Increased urine IgM excretion predicts cardiovascular events in patients with type 1 diabetes nephropathy. BMC Med.

[17] Tofik R, Ekelund U, Torffvit O, Swärd P, Rippe B, Bakoush O (2013). Increased urinary IgM excretion in patients with chest pain due to coronary artery disease. BMC Cardiovasc Disord.

[18] Tofik R, Sward P, Ekelund U (2015). Plasma pro-inflammatory cytokines, IgM-uria and cardiovascular events in patients with chest pain: a comparative study. Scand J Clin Lab Investig.

[19] Smyth A, Oliveira GH, Lahr BD (2010). A systematic review and meta-analysis of pregnancy outcomes in patients with systemic lupus erythematosus and lupus nephritis. Clin J Am Soc Nephrol.

[20] Levey AS, de Jong PE, Coresh J (2011). The definition, classification, and prognosis of chronic kidney disease: a KDIGO Controversies Conference report. Kidney Int.

[21] Levey AS, Stevens LA, Schmid CH (2009). A new equation to estimate glomerular filtration rate. Ann Intern Med.

[22] American College of Obstetricians (2013). Gynecologists, task force on hypertension in pregnancy. Obstet Gynecol.

[23] Verlohren S, Galindo A, Schelbach D (2010). An automated method for the determination of the sFlt-1/PlGF ratio in the assessment of preeclampsia. Am J Obstet Gynecol.

[24] Rana S, Schnettler WT, Powe C (2013). Clinical characterization and outcomes of preeclampsia with normal angiogenic profile. Hypertens Pregnancy.

[25] Leaños-Miranda A, Márquez-Acosta J, Romero-Arauz F (2007). Protein: creatinine ratio in random urine samples is a reliable marker of increased 24-hour protein excretion in hospitalized women with hypertensive disorders of pregnancy. Clin Chem.

[26] Tencer J, Frick IM, Oquist BW, Alm P, Rippe B (1998). Size-selectivity of the glomerular barrier to high molecular weight proteins: upper size limitations of shunt pathways. Kidney Int.

[27] Deen WM, Bohrer MP, Brenner BM (1979). Macromolecule transport across glomerular capillaries: application of pore theory. Kidney Int.

[28] Ota Z, Shikata K, Ota K (1994). Nephrotic tunnels in glomerular basement membrane as revealed by a new electron microscopic method. J Am Soc Nephrol.

[29] Schurek HJ, Neumann KH, Flohr H, Zeh M, Stolte H (1992). The physiological and pathophysiological basis of glomerular permeability for plasma proteins and erythrocytes. Eur J Clin Chem Clin Biochem.

[30] Zhang J-J, Ma X-X, Hao L, Liu L-J, Lv J-C, Zhang H (2015). A systematic review and meta-analysis of outcomes of pregnancy in CKD and CKD outcomes in pregnancy. Clin J Am Soc Nephrol.

[31] Piccoli GB, Fassio F, Attini R (2012). Pregnancy in CKD: whom should we follow and why?. Nephrol Dial Transplant.

[32] Bramham K, Briley AL, Seed PT, Poston L, Shennan AH, Chappell LC (2011). Pregnancy outcome in women with chronic kidney disease: a prospective cohort study. Reprod Sci.

[33] Bharti J, Vatsa R, Singhal S (2016). Pregnancy with chronic kidney disease: maternal and fetal outcome. Eur J Obstet Gynecol Reprod Biol.

[34] Nevis IF, Reitsma A, Dominic A (2011). Pregnancy outcomes in women with chronic kidney disease: a systematic review. Clin J Am Soc Nephrol.

